# Leveraging Aging Service Providers to Support Internet-Based Cognitive Behavioral Therapy for Depression in Homebound Older Adults: Protocol for a Type 1 Hybrid Effectiveness-Implementation Randomized Controlled Trial

**DOI:** 10.2196/72953

**Published:** 2025-09-05

**Authors:** Xiaoling Xiang, Elyse Narbut, Xinyin Zhang, Samson Ash, Skyla Turner, Ruopeng An, Jennifer M Jester, Sunggeun Park, Salma Habash, Joseph A Himle

**Affiliations:** 1 School of Social Work University of Michigan–Ann Arbor Ann Arbor, MI United States; 2 Department of Psychology University of Michigan–Ann Arbor Ann Arbor, MI United States; 3 Silver School of Social Work New York University New York, NY United States

**Keywords:** depression, older adults, homebound, eHealth, mHealth, digital mental health, internet intervention, internet-based cognitive behavioral therapy, iCBT, implementation effectiveness, aging services

## Abstract

**Background:**

Homebound older adults face a high burden of depression and substantial barriers to accessing mental health treatments. Few interventions address their specific needs. Empower@Home, an internet-based cognitive behavioral therapy program, was co-designed with stakeholders and tailored to older adults. The program includes 9 self-paced online sessions to be completed within 12 weeks, augmented by telephone-based human support. Efficacy studies have demonstrated its acceptability and effectiveness in reducing depression when supported by trained research staff. However, its real-world effects and feasibility for integration into community aging service settings remain unknown.

**Objective:**

This study aims to assess the real-world effectiveness of Empower@Home integrated into aging services, using nonclinician agency staff as coaches to support older adults. A secondary objective is to evaluate the implementation process through a qualitative process evaluation.

**Methods:**

The study is a type 1 hybrid effectiveness-implementation trial with a 2-arm randomized controlled design. A total of 256 homebound older adults will be recruited from 3 community aging service agencies, and agency staff will be trained as coaches to support internet-based cognitive behavioral therapy use. Participants in the treatment group will receive Empower@Home immediately, while the control group will receive biweekly friendly calls and enhanced care as usual, including the provision of psychoeducational materials and usual care. Outcomes will be assessed at baseline, after the intervention (12 weeks), and at 2 follow-up points (24 and 36 weeks). The primary outcome is a change in depressive symptoms as measured by the 9-item Patient Health Questionnaire. Secondary outcomes include changes in social isolation, health-related quality of life, disability burden, pain intensity, and anxiety symptoms.

**Results:**

Institutional review board approval was obtained in August 2024, and recruitment began in October 2024. Recruitment is expected to conclude by April 2028. Upon trial completion, the effectiveness of Empower@Home on primary and secondary outcomes will be analyzed.

**Conclusions:**

This study will provide critical insights into the real-world effectiveness of Empower@Home. If successful, this study will provide a scalable, cost-effective model for integrating technology-assisted mental health treatments into community aging services, thereby improving access to care for an underserved, hard-to-reach population.

**Trial Registration:**

ClinicalTrials.gov NCT06584422; https://clinicaltrials.gov/study/NCT06584422

**International Registered Report Identifier (IRRID):**

DERR1-10.2196/72953

## Introduction

### Background

Depression among homebound older adults is a critical yet often overlooked public health issue [1,2]. Research has shown that 50% of homebound older adults experience clinically significant depression. Of these, 14% face major depression, a rate 7 times higher than that of their non–homebound counterparts [3,4]. Regardless of its severity, untreated and undertreated depression has serious health consequences for older adults. It can increase the risk of suicide, physical disabilities, cognitive impairment, hospitalizations, and premature mortality [5-8]. Compounding these challenges, homebound older adults often face substantial access barriers to mental health services due to socioeconomic disadvantages and physical disabilities [9-11].

Late-life depression can be effectively treated with antidepressants, psychotherapies, or a combination of both [12,13]. However, the aging process is often associated with an increased risk of chronic illness, functional limitations, social isolation, and reduced coping resources. Psychotherapies can be particularly useful as they address symptoms and teach coping skills to better address these challenges [10,14]. Unfortunately, few older adults receive psychotherapy treatments due to stigma, a shortage of trained mental health providers, and systemic barriers, including inadequate insurance coverage for subthreshold depression [15-17]. Without proper treatment, depression and physical disability can reinforce each other, ultimately worsening both health and overall quality of life [6].

Aging Network agencies have unmatched reach to the target population, covering nearly every community in the United States and serving more than 8 million older Americans annually. Consequently, integrating mental health treatment into the Aging Network holds significant potential to increase access to mental health services for homebound older adults [10]. Previous research has already demonstrated that nonclinician aging service providers can be effectively trained to deliver a range of depression interventions, including those based on behavioral activation, psychoeducation, problem-solving, and depression self-management [18-25]. For example, Choi et al [26] showed that aging service providers could effectively administer behavioral activation via teleconference [27]. However, depending on these providers to directly administer structured treatments poses several challenges, such as limited expertise in depression care, heavy caseloads, and frequent turnover.

Leveraging digital mental health interventions (DMHIs), particularly internet-based cognitive behavioral therapy (iCBT), is a promising solution to these challenges. When paired with human support, iCBT is as effective as face-to-face CBT [28-30]. Through packaged online lessons, iCBT consistently delivers core therapeutic techniques to patients. Implementing iCBT in aging service agencies shifts providers from direct treatment delivery to supportive roles, thereby reducing training demands and time commitments without compromising CBT fidelity. This makes iCBT a more feasible and scalable approach.

Despite their potential, iCBT programs face significant challenges with user engagement, particularly among older adults. Completion rates have been low, with an average of 17% in self-guided iCBT [31] and 65% in human-support programs [32]. For homebound older adults, engagement barriers are often exacerbated by usability challenges, including text-heavy, academic content and interfaces that are not designed with older users in mind. For example, in our evaluation of an evidence-based iCBT program, only 1 in 4 homebound older adults completed the program, even with weekly in-home support [33]. These findings underscore the urgent need for iCBT programs tailored to older adults.

Although several iCBT programs have been designed to address older adults’ needs, none are widely available to US consumers [34-36]. To address this gap, we developed Empower@Home, an iCBT program explicitly tailored for older adults. Created in collaboration with key stakeholders, the program uses plain language, integrates text with visuals, incorporates age-relevant examples, and features a simplified user interface. Preliminary studies found Empower@Home to have superior usability compared to other iCBT programs. In addition, feasibility trials demonstrated exceptional program completion rates and significant reductions in depressive symptoms when participants were supported by lay coaches [37-40]. However, it remains unclear whether Empower@Home will be equally effective when integrated into real-world community aging services and supported by aging service providers.

### Objective

The primary aim of this study is to evaluate the real-world effectiveness of Empower@Home, an iCBT program tailored to older adults with depression when supported by aging service providers. A mixed methods approach will be used, incorporating structured surveys and semistructured interviews to examine intervention outcomes and underlying mechanisms. The secondary aim is to identify implementation barriers and facilitators through a qualitative process evaluation. This study builds on previous efficacy trials demonstrating the acceptability and effectiveness of Empower@Home. It takes a critical next step by transitioning the intervention from an academic setting into community-based aging services, thereby assessing its real-world impact and the feasibility of training aging service providers to act as coaches or human supporters for iCBT.

### Intervention Model

The design of Empower@Home incorporates user-centered and community-participatory research principles [37]. The intervention was designed with input from homebound older adults, therapists, and more than 30 aging service providers, undergoing multiple iterative refinements based on testing and feedback [37-39]. Empower@Home consists of 9 sequenced online lessons, each lasting 20 to 25 minutes, featuring didactic content delivered through short videos, voice-over instructions, interactive exercises, and motivational quotes. These lessons are grounded in evidence-based CBT manuals [41-43] and focus on core CBT skills: “Doing Tools” for behavioral activation and problem-solving, “Thinking Tools” for cognitive restructuring, “Feeling Tools” for relaxation and mood monitoring, and “Communication Tools” for effective communication [41].

The content is specifically tailored to the unique needs of homebound older adults, incorporating age-relevant examples informed by stakeholder input and prior research [44-46]. Each lesson integrates a mini animated story centered on a homebound older woman, Jackie, facing common health challenges within the target demographic. According to persuasive design principles, interactive, engaging, and immersive interventions are more effective in promoting behavioral change [45]. Entertainment education theories further suggest that narratives can reduce resistance to behavioral modifications by fostering emotional engagement and personal reflection [46]. Jackie’s story encourages users to identify with the character, adopt story-aligned behaviors, and improve treatment engagement. Qualitative interviews with past participants indicated mixed identification with the storyline but suggested that it enhanced engagement [40].

Aligned with best practices for age-friendly digital design [47], the program features a streamlined interface with large buttons, text-labeled icons, high-contrast colors, and intuitive navigation. Online lessons are complemented by a large-print workbook containing session summaries, additional psychoeducational materials, wellness resources, and home practice instructions. Designed with older adults in mind, the workbook features large text, images of diverse older adults, and a visually appealing layout. Prior studies highlighted the workbook’s importance, noting its familiar format, aesthetic appeal, and utility as a convenient reference for completing and revisiting exercises and lessons [40].

To address real-time support needs and technological challenges, we introduced “Empower Coaches,” laypersons who provide weekly support calls to enhance program personalization, guided by the Efficiency Model of Support [37]. Our target demographic favors this human interaction over purely automated systems. Supported iCBT programs consistently demonstrate better engagement and clinical outcomes than those without support [30-32]. While many iCBT programs rely on therapists with specialized mental health qualifications, using aging service providers as iCBT supporters offers an innovative and practical solution. This strategy leverages aging service providers’ existing relationship with the target population and aligns with the staffing structures of aging service agencies, ensuring widespread and accessible support. Prior research has successfully trained aging service providers to administer psychotherapy and multicomponent interventions directly [18-25], suggesting that training aging service providers as coaches is also likely feasible.

Our approach focuses on equipping service providers to support iCBT, a role that does not require extensive specialized training and is less time-intensive than acting as lead interventionists. Our previous efficacy studies showed that Empower@Home, supported by undergraduate and graduate students in psychology, social work, or related fields, was acceptable and efficacious based on both qualitative and quantitative outcomes [38,40]. However, no studies have examined the feasibility of training aging service providers such as case workers and community health workers to support iCBT or integrating iCBT into aging service settings. This study aims to fill that gap.

Our team developed a structured coaching guide based on experiences from pilot studies. The guide outlines coaching tasks, provides tips, and includes CBT teaching points aligned with session content. Coaches conduct weekly check-ins, which can be completed over the phone or in person, allowing flexibility and greater efficiency. For example, coaches with scheduled home visits can incorporate coaching sessions into the visit without requiring additional scheduling. On the basis of prior research and our experience, coaching support typically includes providing motivation, encouragement, homework review, assistance with applying tools, and technical support. In line with the efficiency model of support [48], coaches are encouraged to personalize their approach for each client—for instance, offering to complete part of the program alongside an unmotivated participant. The length of coaching sessions can be adapted to meet participants’ needs and preferences, ranging from 5 minutes to an hour.

Coach training includes reviewing all Empower@Home online sessions (approximately 4-5 hours), supplemented with didactic videos introducing each session and its techniques. In addition, coaches attend a live, in-person training workshop lasting approximately 4 hours, where they observe demonstrations of coaching sessions and receive clarification on operating logistics. The total training requirement is approximately 9 to 10 hours per coach. Biweekly coach support meetings are offered throughout the study to provide ongoing support. While previous studies limited the intervention period to 10 weeks, this project extends it to 12 weeks to accommodate the frailty of the target population, who may face additional barriers such as hospitalizations that could delay program completion.

## Methods

### Preliminary Study

The procedures outlined in this protocol, including recruitment, assessments, coaching call logistics, and device management, have been tested and refined through feasibility and efficacy studies conducted in 2022 to 2023. A pilot randomized controlled trial (RCT) with 70 participants, supported by trained research assistants, demonstrated that the program was significantly more effective in reducing depressive symptoms compared to biweekly friendly calls, with a medium between-group effect size (Cohen *d*=0.7) [38]. Program adherence was high, with approximately 90% of participants completing all sessions within 10 weeks [39]. Participants reported high satisfaction with the program, and themes from qualitative interviews supported the quantitative findings, further highlighting the program’s positive effects [40]. However, positive findings from small-scale pilot studies may not always translate into comparable outcomes in real-world settings or larger studies. This trial evaluates whether these promising results can be replicated in a community-based, real-world implementation context.

### Study Setting

The University of Michigan will be the primary study site, collaborating with community agencies serving older adults. All participants will be ≥60 years and are expected to be predominantly low-income individuals. They will complete the intervention at home, with all assessments and technological support provided remotely by phone.

### Eligibility Criteria

To participate in the trial, individuals must be at least 50 years old, eligible to receive services at the participating community agency, and have mild or greater depressive symptoms, defined as a score of ≥8 on the 9-item Patient Health Questionnaire-9. Participants will be excluded if they meet any of the following criteria: (1) probable cognitive impairment, indicated by a score of ≥12 on the Blessed Orientation-Memory-Concentration Test [49]; (2) non-English speakers; (3) moderate to high suicide risk, as assessed by the Columbia-Suicide Severity Rating Scale [50]; (4) terminal illness with a life expectancy of fewer than 6 months or unstable physical health; (5) visual impairment that prevents the use of a screen, even with corrective measures (self-reported); (6) current or recent substance use, as indicated by a score of ≥2 on the CAGE-Adapted to Include Drugs Substance Abuse Screening Tool [51]; (7) diagnosis of a psychotic disorder (self-reported); and (8) active psychotherapy (more than once a month) or initiation of new therapy within the past 3 months. The study will not exclude participants based on race or gender. While completing the intervention requires a tablet and internet access, participants are not required to have their own. Those without a tablet or internet access will be provided with both at no cost for the active intervention period.

### Recruitment and Compensation

Participants will be recruited on a rolling basis, primarily through referrals from participating community agencies. Two of these agencies are Area Agencies on Aging (AAAs), which may refer existing clients enrolled in their programs, such as case management, caregiver services, or Meals on Wheels. In addition, AAAs may refer new clients or individuals seeking information from the agency, as providing information is a key role of AAAs.

To support participant recruitment, participating agencies receive a 20-minute online training covering eligibility criteria and referral procedures. The research team also designed a prescreening form that includes the PHQ-9 and additional brief screening items to improve referral accuracy. On the basis of prior experience, approximately half of the individuals passing the prescreening will qualify for the study. All prescreening and referral forms are hosted online and optimized for efficiency.

After receiving referrals, study personnel will contact potential participants by phone to complete a more detailed eligibility screening. This initial screening typically takes 20 minutes but may take longer if a complete suicidal ideation assessment is needed. Participants who agree to participate will provide recorded verbal consent, while those who decline will be referred back to their case managers or agency when feasible. In addition, participants can self-refer for eligibility screening through advertisements on social media platforms and by word of mouth.

Qualified participants will be compensated up to US $140, including US $30 for each survey assessment and US $20 for the qualitative interview. Compensation is prorated based on completed assessments (eg, participants completing only the baseline assessment will receive US $30).

### Study Design and Randomization

Eligible and consented participants will be randomly assigned to the treatment or control group in a 1:1 ratio. Block randomization, stratified by participating community agencies, will be used to assign participants. Each site has 9 blocks of 10 participants. The study statistician (JNJ) created the randomization sequence, and sealed envelopes were prepared by a research assistant who was not involved in the randomization process. The lead project coordinator will conduct the randomization using these sealed envelopes.

Both groups will follow the same assessment schedule, including a comprehensive baseline assessment, a postintervention or control assessment at 12 weeks, and follow-up assessments at 24 and 36 weeks. Both groups will continue to receive usual care from their referring agency. Participants in the control group will receive the intervention after completing their final follow-up assessment. Due to the nature of the intervention and study design, blinding participants and evaluators to group assignments is not feasible. However, assessors will remain blinded to group allocation whenever possible.

### Control Condition

The attention control condition provides participants with a comparable amount of phone contact as the intervention group, including 15- to 25-minute companionship calls (“friendly calls”) every other week. These calls are conducted by research team members and focus on providing social interaction and support without introducing CBT-related content or structured therapeutic interventions. To ensure safety and align with the biweekly PHQ-9 assessments conducted in the intervention group as a part of the online program, participants in the control group will complete a telephone-administered PHQ-9 assessment every 2 weeks as a part of the friendly calls. In addition, participants in the control group will continue receiving regular social services from the participating agency. They will receive a psychoeducational handout containing information about depression and a list of community resources for further support.

### Safety Monitoring and Discontinuation

Participant safety is a top priority in this study, with procedures in place to monitor and address any significant clinical changes. Biweekly assessments of depressive symptoms using the PHQ-9 serve as both clinical outcome measures and safety checks. A clinically significant worsening of symptoms, defined as a 5-point or greater increase in the PHQ-9 from baseline, triggers a multistep review process. This process includes automated alerts in our electronic data-capturing system and in-depth risk assessments by the project coordinator, a licensed clinical social worker. While such occurrences are rare—less than 3% of participants experienced a 5+ point increase during pilot studies—persistent or worsening symptoms over 4 weeks may lead to discontinuation. Similarly, any new or increased endorsement of suicidal ideation on item 9 of the PHQ-9 prompts immediate follow-up using the Columbia-Suicide Severity Rating Scale, following an established protocol to ensure participant safety.

Participants may also be withdrawn from the study if their physical health conditions worsen unrelated to the intervention, compromising their ability to participate safely (eg, repeated hospitalizations). Such decisions are informed by input from the participant, agency coaches, and family members. In all cases of withdrawal, the study team collaborates with partnering community agencies to connect participants to appropriate treatment, including pharmacotherapy and locally available psychosocial resources. Final discontinuation decisions will be made by the core research team managing human participants, including the principal investigator and the 3 project staff.

### Data Management and Storage

All study data collected through surveys or structured observations will be entered directly by project staff into REDCap (Research Electronic Data Capture; Vanderbilt University) [41]. Agency coaches will also complete a structured coaching form hosted on REDCap. During the active intervention, participants will complete the PHQ-9 every other week as part of the online therapy lessons. These assessment data will be securely stored in a HIPAA (Health Insurance Portability and Accountability Act)–compliant database hosted by the University of Michigan. Recordings of qualitative interviews will be stored on Dropbox in compliance with the University of Michigan’s safe computing guidelines for handling sensitive research data. In addition, meeting minutes and other program administrative records, which do not contain participant identifying information, will be stored on Google Drive.

### Measures

#### Primary Clinical Outcomes

**Figure 1:**
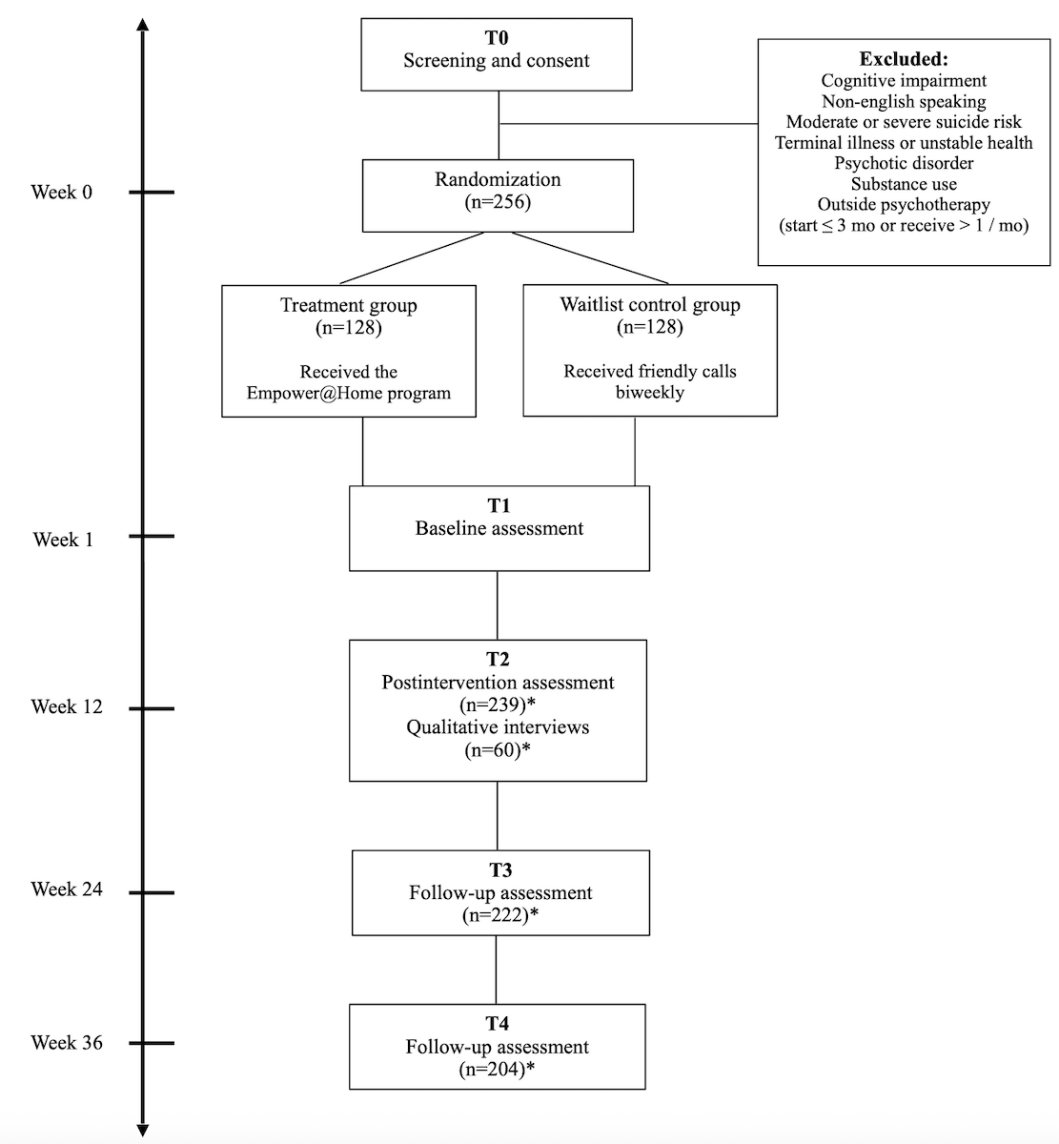
Participant flow. *Accounting for a cumulative 20% attrition rate from baseline to T4.

All key outcome measures are summarized in Table 1. The primary outcome is changes in depressive symptoms, assessed using the PHQ-9, a widely used screening tool in primary care, community agencies, and iCBT studies [52]. Each participant will complete the PHQ-9 up to a total of 9 times: at screening (T0), postintervention at 12 weeks (T2), and follow-up assessments at 24 weeks (T3) and 36 weeks (T4), along with up to 5 biweekly assessments between baseline (T1) and postintervention period (T2). For participants in the intervention group, PHQ-9 assessments will be embedded within the online program and completed during sessions 1, 3, 5, 7, and 9. Control group participants will complete these assessments via telephone at corresponding intervals. In addition, the 7-item Hamilton Depression Rating Scale (HAMD; HAMD-7) will be used as a secondary measure to evaluate the intervention’s clinical effectiveness. The HAMD-7, an abbreviated version of the HAMD-17, is shown to be equally effective in assessing remission in patients with major depressive disorder [53]. It was chosen to reduce participant burden and to enhance the feasibility of telephone-based administration. The participant flowchart is presented in Figure 1.

**Table 1:** Key study measures and time points.

Measurement domain		Instrument	Baseline (T1)	Biweekly	Post (T2)	Follow-up (T3)	Follow-up (T4)
**Primary clinical outcomes**							
	Depressive symptoms	PHQ-9^a^	✓	✓	✓	✓	✓
	Depressive symptoms	HAMD-7^b^	✓		✓	✓	✓
**Secondary clinical outcomes**							
	Anxiety	GAD-7^c^	✓		✓	✓	✓
	Social isolation	DSSI-10^d^	✓		✓	✓	✓
	Health-related quality of life	EQ-5D-5L	✓		✓	✓	✓
	Disability burden	WHODAS 2.0^e^	✓		✓	✓	✓
	Pain	PEG^f^	✓		✓	✓	✓
**Treatment mechanisms**							
	CBT^g^ skills acquisition	CBTSQ^h^	✓		✓		
	Behavioral activation	BADS-SF^i^	✓		✓		
	Dysfunctional attitudes	DAS-SF2^j^	✓		✓		
	Social engagement	SEAQ^k^	✓		✓		
	Psychological flexibility	AAQ-II^l^	✓		✓		
	Digital alliance	D-WAI^m^			✓		
	Narrative engagement	NES^n^			✓		
	Character identification	EDI^o^			✓		
	Coaching fidelity	Study-specific			✓		
	Autonomy	BNSQ-S^p^	✓		✓		
**Feasibility and acceptability**							
	Treatment adherence	Session completion			✓		
	Treatment acceptability	TEI^q^			✓		

^a^PHQ-9: 9-item Patient Health Questionnaire.

^b^HAMD-7: 7-item Hamilton Depression Rating Scale.

^c^GAD-7: 7-item Generalized Anxiety Disorder.

^d^DSSI-10: 10-item Duke Social Support Index.

^e^WHODAS 2.0: WHO Disability Assessment Schedule 2.0.

^f^PEG: Pain, Enjoyment of Life and General Activity.

^g^CBT: cognitive behavioral therapy.

^h^CBTSQ: Cognitive-Behavioral Therapy Skills Questionnaire.

^i^BADS-SF: Behavioral Activation for Depression Scale–Short Form.

^j^DAS-SF2: Dysfunctional Attitudes Scale–Short Form 2.

^k^SEAQ: Social Engagement and Activities Questionnaire.

^l^AAQ-II: Acceptance and Action Questionnaire.

^m^D-WAI: Digital Working Alliance Inventory.

^n^NES: Narrative Engagement Scale.

^o^EDI: Identification with Characters Scale.

^p^BNSQ-S: Basic Needs Satisfaction in General Scale.

^q^TEI: Treatment Evaluation Inventory.

#### Secondary Clinical Outcomes

The secondary clinical outcomes, listed in Table 1, include changes in anxiety, social isolation, quality of life, disability burden, and pain severity, assessed at 4 time points (T1-T4). These outcomes were chosen for their significant impact on the quality of life of older adults with chronic illnesses and disabilities, aligning with prior research on geriatric depression treatments [20,54].

Anxiety will be assessed using the Generalized Anxiety Disorder-7, a measure that has been validated in older adults [33,55]. Participants answer 7 questions probing the frequency of their anxiety symptoms rated on a 4-point Likert scale ranging from “not at all” to “nearly every day.” The Generalized Anxiety Disorder-7 scale has demonstrated 89% sensitivity and 82% specificity in detecting generalized anxiety disorders in primary care patients [37].

Social isolation will be assessed using the Duke Social Support Index 10-item scale, which evaluates social interaction and satisfaction with social support in older adults [56].

Quality of life will be measured using the EQ-5D-5L scale [57]. Participants rate their ability to perform tasks in 5 domains—mobility, self-care, usual activities, pain, and anxiety or depression—on a 5-point Likert scale ranging from “no problem” to “extreme problem or unable.” Responses will be converted into utility values using a US population–specific algorithm [58].

Disability burden will be assessed using the WHO Disability Assessment Schedule 2.0 12-item survey, which examines difficulty performing activities across 6 domains: cognition, mobility, self-care, getting along with people, life activities, and societal participation. Responses range from “none” to “extreme or cannot do” [59].

Pain severity will be evaluated with the Pain, Enjoyment of Life and General Activity scale, a 3-item measure that assesses pain intensity and its interference with enjoyment of life and general activity. Each item is rated on a scale from 0 to 10 [60].

#### Treatment Mechanisms

##### Overview

We hypothesize that the intervention operates through multiple mechanisms grounded in CBT, entertainment persuasion, and self-determination theories. CBT principles suggest that depression stems from maladaptive thoughts and behaviors; thus, improved CBT skills, behavioral activation, and reduced dysfunctional attitudes are expected to mediate treatment effects [61]. The animated Jackie storyline aims to enhance engagement, fostering a connection with the narrative and program [46]. In addition, the coach-client rapport, aligned with the common factors theory, is hypothesized to mediate outcomes through the therapeutic bond and satisfaction with coaching [62]. Self-determination theory highlights the importance of autonomy, competence, and relatedness in fostering motivation and well-being [63].

##### CBT-Related Factors

Jacob et al [64] developed the 16-item Cognitive-Behavioral Therapy Skills Questionnaire to assess the use of CBT skills such as cognitive restructuring and behavioral activation. We modified the scale to 14 items by removing 2: “Identify stressors that led me into treatment,” as participants were not recruited from clinical settings, and “Catch myself when I jump to conclusions,” as the preprogram assessment occurs before participants are introduced to this concept, enhancing reliability. Item wording for the remaining 14 items was simplified for readability, for example, “Identify risk factors for relapse” was revised to “Identify warning signs that trigger bad mood, depression, or anxiety.” Participants rate their frequency of activities such as organizing plans, motivating themselves, and socializing on a 5-point Likert scale from “never” to “always.”

The 9-item Behavioral Activation for Depression Scale–Short Form evaluates activation and avoidance behaviors consistent with Behavioral Activation theory [65]. Each item is rated on a 7-point scale from 0 (“not at all”) to 6 (“completely”), with higher scores reflecting greater activation.

The 9-item Dysfunctional Attitudes Scale–Short Form 2 is a self-report measure for assessing the presence and intensity of rigid or maladaptive beliefs [66]. We adapted the original 4-point Likert scale (“totally agree,” “agree,” “disagree,” and “totally disagree”) to a 5-point Likert scale ranging from “strongly disagree” to “strongly agree” to be consistent with the other measures in the study.

The 10-item Social Engagement and Activities Questionnaire was developed to assess social engagement in homebound older adults, accounting for physical and functional limitations. Participants report the frequency of activities such as socializing, volunteering, attending religious services, and participating in recreational or educational events over the past month. Responses are scored on a 6-point scale (0-5) for each item [67].

##### Entertainment or Engagement-Related Factors

The Digital Working Alliance Inventory is a 6-item measure that asks participants to rank their experience using a DMHI [68]. Responses are scored using a 5-point Likert scale from “strongly agree” to “strongly disagree.” The word “app” from the original Digital Working Alliance Inventory scale was modified to “program” to reflect that the intervention is accessed online rather than through a mobile app.

The 12-item Narrative Engagement Scale assesses the audience’s mental models, particularly their enjoyment and understanding of a story, using a 5-point Likert scale ranging from “strongly agree” to “strongly disagree” [69]. The scale includes 4 domains, each measured by 3 items: narrative understanding, attentional focus, narrative presence, and emotional engagement. For this study, we retained all 3 items from the emotional engagement domain but selected only one item each from the narrative understanding, attentional focus, and narrative presence domains.

The Identification with Characters Scale consists of 14 items that help identify different levels of relatedness and connectedness the viewer has to a film [70]. We adapted the original 5-point scale (“1 = not at all” to “5 = very much”) to a 5-point scale ranging from “strongly disagree” to “strongly agree” for consistency with other questions. In addition, “characters” was replaced with Jackie’s name (eg, “I felt like I was similar to Jackie”).

##### Coach-Related Factors

Coaching fidelity (client report) will be assessed using a 9-item scale developed specifically for this study. Some items were adapted from the Therapist’s Interpersonal Style Scale [71] to align with the study context, emphasizing aspects of coaching that reflect therapeutic alliance and its potential influence on treatment outcomes. Example items include “The feedback I receive from my coach is helpful,” “My coach is trustworthy,” and “My coach makes the program more personalized and relevant to my situation and needs.” Participants will rate their agreement with each statement on a 5-point Likert scale ranging from “strongly agree” to “strongly disagree.”

##### Self-Determination

The Basic Needs Satisfaction in General Scale is a 16-item questionnaire designed to assess satisfaction with 3 fundamental psychological needs: autonomy, competence, and relatedness. Autonomy pertains to an individual’s desire to perceive their actions and subsequent consequences as self-determined, rather than being subject to external influences or control [72]. Competence refers to the inherent desire to experience a sense of efficacy and proficiency in executing tasks of varying complexities [73]. Relatedness refers to the inherent human desire for social connection, support, and caring from others [73]. According to the self-determination theory, all 3 needs must be fulfilled to achieve psychological well-being [72]. Consistent with the responses of other measures used in this study, participants respond on a 5-point scale ranging from “strongly agree” to “strongly disagree” [74].

Acceptability will be assessed by the number of sessions participants completed (ie, adherence) and the Modified Treatment Evaluation Inventory (TEI) developed explicitly for evaluating geriatric depression treatment [75]. The TEI has 11 items, including 8 positively worded items and 3 negatively worded items. We modified the TEI by rephrasing the original TEI items from questions to statements and reducing the responses from a 7-point Likert scale to a 5-point Likert scale to ease respondents’ burden. The modified TEI yields total scores ranging from 11 to 55, where a score of ≥32 suggests positive attitudes toward the treatment.

#### Implementation Fidelity Monitoring

Implementation fidelity is essential for evaluating interventions delivered in real-world settings. While the online program ensures consistent delivery of core CBT concepts, coaching support naturally varies due to its personalized nature. We used 3 methods to assess fidelity in this context: coach self-reports, in vivo observations of coaching calls, and fidelity check-in calls with clients.

Coach self-reports capture the coach’s perspective on each session. Coaches document session duration, delivery mode (phone, virtual, or in-person), and types of support provided, selecting from a checklist including application, education, motivation, personalization, and technology assistance. In addition, the coaches respond to close-ended questions assessing adherence to session tasks outlined in the coaching guide, such as “Did you review in-session exercises as outlined in the coaching guide?” with response options of all, most, some, or none.

In vivo observations involve trained research team members evaluating at least one coaching session per client, selected randomly using a predetermined random sequence. Observers join sessions via 3-way calls to assess fidelity in real time. A structured rating guide, developed specifically for this study, includes items adapted from the literature and validated scales such as the United Kingdom Alcohol Treatment Trial Process Rating Scale [76] and the Therapist’s Interpersonal Style Scale [71]. The guide comprises items across 3 domains: session management, adherence to the coaching guide, and coaching style. Key items under session management and adherence to the coaching guide include collaborative agenda-setting, reviewing exercises, checking goal progress, and providing personalized feedback. Coaching style is rated based on factors such as engagement, empathy, reflective listening, and collaboration. Each item is scored on a 4-point scale from excellent to poor. The instrument includes detailed instructions for rating each item. To ensure reliability, 2 research staff independently rated coaching call recordings from pilot studies, compared their ratings, and resolved discrepancies to refine the scale and its instructions for consistency.

#### Qualitative Interviews

After completing the intervention, approximately half of the intervention group will take part in a semistructured qualitative interview lasting approximately 30 minutes. These interviews will explore several domains, including participants’ overall experience with the program, engagement barriers and facilitators, and their experience with the coaching component. Interview questions are available in Multimedia Appendix 1.

In addition, all Empower Coaches, their immediate supervisors (who also serve as site principal investigators for institutional review board [IRB] purposes), and other relevant agency staff involved in implementation will be invited to participate in qualitative interviews during the final year of data collection. These interviews will gather insights into how the intervention functioned, what modifications were or should be made, and how well it integrates into routine provider workflows. The updated Consolidated Framework for Implementation Research [77] will guide the design of qualitative interview protocols and data analysis.

### Sample Size

All parameters for the power analysis were based on the pilot RCT conducted in 2023, with an original sample size of 70, with 35 in the treatment group and 35 in the control group. At the time of this analysis, a total of 440 data points across the 7 time points had been collected. Time 7 data had been collected from 63 participants, 33 in the treatment and 30 in the control group. A linear mixed model using *lme* from R package *lme4* showed a treatment by time effect of −0.69, *P*<.001. These pilot data were used in the R package *simr* with the effect size conservatively set to approximately one-half of that found in pilot study, −0.35. With a sample size of 192, there was a power of 0.91 to detect the effect of −0.35 for the treatment by time parameter. Assuming 25% attrition, we will aim to recruit 256 participants.

### Analytic Plan

#### Primary Analyses

The primary analyses will use an intention-to-treat approach, including all individuals who completed the baseline interview and were randomized. If sufficient variability in participation or dropout is observed, secondary analyses will incorporate measures of intervention dosage (ie, adherence) as covariates to account for differences in engagement with the intervention.

Linear mixed effects models will be used to examine differences between the treatment and control groups over time. All outcome variables will be assessed for normality, and transformations will be applied if needed. Parameter estimates for the interaction between group membership and time will determine whether the treatment group demonstrates significantly different changes over time compared to the control group. Various covariance structures will be evaluated to identify the best-fitting model, and nonlinear time functions (eg, quadratic) will also be explored.

#### Mediation Analysis

Latent growth curve modeling will be used to assess whether proposed mediators—CBT-related, engagement-related, and coach-related factors—explain the primary treatment effect [78]. These models will be estimated using Mplus software. According to Cheong [79], latent growth curve models with 5 measurement points show minimal bias and sufficient power to detect mediation effects, particularly when the *R*² (the proportion of variance in mediator and outcome variables explained by growth factors) is moderate or high. In this study, 7 measurement points will be used in mediation analysis (T1, T2, and 5 in-app assessments), making the estimates conservative as power increases and bias decreases with additional measurements. On the basis of guidance from Cheong [79], assuming a medium effect size and moderate *R*², the sample size of 192 will yield power of 0.8 to detect the mediation effect, indicating adequate statistical power for the analysis.

#### Qualitative Analysis

Third-party transcription services will be used to transcribe recorded qualitative interviews. Coding and theme extraction will follow the methodology proposed by Saldaña [80] for qualitative analysis. In the first coding cycle, a combination of a priori codes from preliminary studies and inductively developed new codes will be used to analyze transcripts. Two trained research staff will independently review a random subset of transcripts to ensure coding consistency. This process will continue until a satisfactory level of agreement is reached, at which point, the finalized codebook will be applied to the remaining transcripts. During the second coding cycle, patterns and relationships among initial codes will be identified, with codes being grouped into broader themes or concepts through an iterative process. Data analysis will be conducted using Dedoose, which supports essential qualitative analysis tasks such as coding, creating memos, and diagramming.

### Ethical Considerations

The study protocol was approved by the University of Michigan Health Sciences and Behavioral Sciences IRB (HUM00254688). Participants provide verbal consent, which is recorded after receiving information about the study’s purpose and potential outcomes. Written consent was waived because the study does not involve in-person contact and would not require written consent outside of the research context. After verbal consent, a written copy of the informed consent is mailed to participants. If a participant opts out of recording consent, they receive the consent form by mail along with return postage to send back the signed document. Participants receive compensation for their time completing the study surveys, up to US $140 in total paid via check or gift card. Participant contact information is kept for the purposes of follow-up during participation and is kept separately from study data using REDCap, a HIPAA-compliant secure platform. Deidentified study data will be uploaded to the National Institute of Mental Health Data Archive, in accordance with the sponsor’s requirements. Participants are asked for their consent to retain their deidentified study data after the study conclusion. After the research data are verified and all payments have been processed to participants, identifiable data will be deleted within 6 months of the study conclusion and research records of those who consented will be kept as a deidentified dataset.

## Results

Funding for this project was awarded in July 2024. IRB approval was obtained in August 2024. Enrollment began in October 2024 and is anticipated to be completed in April 2028. The trial is registered at ClinicalTrials.gov (NCT06584422).

## Discussion

### Anticipated Findings

To our knowledge, this study represents the first hybrid type 1 implementation-effectiveness RCT of an aging service provider–supported iCBT intervention for low-income homebound older adults with depression. We hypothesize that the Empower@Home intervention will reduce depressive symptoms and improve psychosocial functioning and health-related quality of life when supported by aging service providers.

Despite the availability of numerous iCBT programs, very few are explicitly designed for older adults [35,36,81,82], a population more sensitive to usability challenges. Our previous experience testing generic iCBT programs with low-income homebound older adults revealed numerous usability issues, such as hard-to-read text, buttons placed too close together, icons without labels, required text entry fields, and complex interfaces, leading to frustration and low program completion [33,34]. Even college-educated and computer-literate older adults report challenges with technology, such as text entry, when interacting with iCBT programs [83]. Having a streamlined, user-friendly, and accessible user interface is critical to the experience of older adult consumers. In addition to user interface, experts have articulated the need for procedural and content modifications to CBT that address differences in thinking styles and age-related adjustment [84]. In our previous study, older adult users described a general adult iCBT program as, “This isn’t meant for someone my age,” due to the lack of relatable case stories or examples that reflected their life experiences [44]. Furthermore, the real-world effectiveness of these programs has often been limited by difficulties in achieving consistent and meaningful user engagement outside of academic settings [85]. This study addresses these gaps by tailoring an iCBT program specifically for older adults and embedding it within community-based aging services supported by trained aging service providers.

This study makes 2 significant contributions to the field. First, it provides crucial evidence on integrating iCBT with training community providers, such as aging service staff, to offer human support. This approach tests the feasibility and effectiveness of using providers already embedded in the community to deliver iCBT in real-world settings. While our prior efficacy trials demonstrated that layperson-supported iCBT can succeed in highly controlled academic environments, this study explores whether those results can be replicated in community settings.

Using nonclinician providers has been recognized as a key approach to expand the geriatric mental health workforce [16]. Leaders in geriatric mental health have demonstrated that nonclinician aging service providers can be trained to effectively provide behavioral interventions for depression [20-25]. However, none of the previous studies tested DMHIs. In addition to filling the gap regarding aging service staff–supported DMHIs, this study also provides importance insights into implementation issues, which remains an underexplored area of research. We will gather information on the implementation process and address questions about treatment fidelity. Specifically, we will examine whether aging service providers can consistently follow the intervention protocol and quality of coaching. We anticipate larger variation in implementation fidelity in this real-world effective study than that observed in our previous studies with student coaches in a controlled academic setting. Interestingly, the intergenerational aspect of using student coaches in past trials may have played a unique role that providers with existing relationships with their clients might not replicate. Therefore, it is important to verify that the program will be effective in real-world settings. The findings will lay the groundwork for future large-scale implementation and scalability efforts.

Second, our previous efficacy trials showed exceptional program engagement, with 90% of participants completing the program—a stark contrast to the average completion rates reported in meta-analyses. For example, one meta-analysis of 40 iCBT studies found that 57% of participants dropped out [86]. More recent systematic reviews have shown that completion rates average 17% for self-guided DMHIs [87] and 65% for therapist-supported interventions [32]. In addition, use rates often drop sharply when these interventions are implemented outside controlled study conditions [86,88]. While our earlier qualitative studies offered insights into high engagement [40], many factors contributing to this success remain unknown. In this study, we will examine engagement-related, coaching-related, and CBT-related mechanisms to better understand treatment pathways. These findings could provide broader insights into overcoming engagement challenges in DMHIs.

The rapid evolution of digital mental health technologies, particularly with advancements in artificial intelligence, underscores the importance of addressing the digital divide. Without careful consideration, these innovations risk further marginalizing underserved populations, including low-income older adults. This study demonstrates how technology can be leveraged alongside existing service delivery infrastructures to address unmet mental health needs. It provides a critical data point on effectively integrating DMHIs into community settings effectively.

Finally, this study is particularly timely given the long-term mental health consequences of the COVID-19 pandemic [89], which have disproportionately affected vulnerable populations such as homebound older adults. By addressing engagement and implementation challenges, this research contributes to the ongoing effort to close mental health disparities and ensure equitable access to effective treatments.

### Strengths and Limitations

This study has several notable strengths. The intervention was iteratively developed, incorporating patient and provider feedback, and was tested using a rigorous randomized design. It uses laypersons as interventionists and is implemented in real-world community settings, thus enhancing its practical relevance. The study includes a 36-week follow-up period to assess long-term effects and focuses on an underserved, hard-to-reach population with significant unmet mental health needs.

However, the study also has limitations. While it is fully powered at the individual participant level to detect both treatment effects and mediation effects, it is not powered to detect differences at the coach or site level. Our plan includes only 3 community sites, and each site is expected to have 2 coaches. Preliminary analyses of the first 50 enrolled participants already show variation in participants’ backgrounds across sites. These differences may stem from multiple factors, including regional variation (eg, Metro Detroit vs a predominately rural county), differences in clientele given agencies’ priorities, and methods agency staff use to identify clients. For example, we have observed initial patterns in baseline depression burden across sites, likely influenced by agency-specific outreach and referral strategies. In addition to differences in clientele, we have also observed qualitative differences across sites in organization culture, leadership style, communication, and internal processes (eg, incentives for coaches and allocation of protected time for coaching). These factors may influence how coaching is delivered and the fidelity of the intervention implementation across sites. To rigorously evaluate these contextual factors, future studies should adopt implementation-focused designs with a larger number of community organizations and randomization and the site level, if comparing different implementation strategies. Similarly, coach-level differences, such as variations in qualifications and experiences, may also affect coaching fidelity. However, in this study, coach assignment within each site is not randomized. Instead, clients are matched to coaches based on a combination of preference and convenience. For example, if coach A referred a client, coach A is typically assigned to coach that client. In other cases, the assignment may depend on coach availability—such as assigning coach B because they have time to take on a new client. As a result, coach-level differences are confounded by client-level characteristics, making it impossible to draw causal conclusions about the effectiveness of individual coaches. To accurately assess coaching effectiveness across different coaches, clients would need to be randomly assigned to coaches in future studies.

Another limitation to consider is the difference in how the PHQ-9 is administered between the treatment and waitlist groups during the intervention period. Treated participants complete the PHQ-9 on their own through the online program platform (self-administered). In contrast, waitlisted participants complete the PHQ-9 over the phone with research staff, as they do not have access to the online platform. This introduces a systematic difference in the mode of administration, which could potentially influence how participants respond and score on the PHQ-9. Importantly, this difference only affects the repeated PHQ-9 scores used in the mixed effects models. It does not impact our primary outcome analysis, which is based on the change from baseline to posttest scores, both of which are administered verbally by trained research staff for all participants, regardless of group. To further assess whether the mode of administration introduces bias, we will compare participants’ baseline PHQ-9 scores (administered by staff) with their in-app self-assessment scores.

### Conclusions

This study offers some of the first evidence on integrating DMHIs into aging services. If successfully implemented, this innovative approach could help bridge the digital divide and address mental health disparities among homebound older adults. In addition, the findings have broader relevance for other DMHIs and populations with physical disabilities or access barriers who continue to face significant barriers to mental health care.

## Appendix

Multimedia Appendix 1 Interview guide.

Multimedia Appendix 2 CONSORT-EHEALTH (V 1.6.1) checklist.

Multimedia Appendix 3 Peer review report from the EMHI - Effectiveness of Mental Health Interventions Study Section, National Institute of Mental Health Initial Review Group. National Institute of Mental Health (National Institutes of Health, USA).

## Abbreviations

AAAs: Area Agencies on Aging
DMHI: digital mental health intervention
HAMD: Hamilton Depression Rating Scale
HIPAA: Health Insurance Portability and Accountability Act
iCBT: internet-based cognitive behavioral therapy
IRB: institutional review board
PHQ-9: 9-item Patient Health Questionnaire
RCT: randomized controlled trial
REDCap: Research Electronic Data Capture
TEI: Treatment Evaluation Inventory

## Data Availability

The datasets generated during this study will be available in the National Institute of Mental Health Data Archive (NDA) repository.
