# Supplementary material for: Leveraging Aging Service Providers to Support Internet-Based Cognitive Behavioral Therapy for Depression in Homebound Older Adults: Protocol for a Type 1 Hybrid Effectiveness-Implementation Randomized Controlled Trial
Source: JMIR Res Protoc. 2025 Sep 5;14:e72953. doi: 10.2196/72953 (PMC12449670; doi:10.2196/72953)
Supplement: Multimedia Appendix 1 [file resprot_v14i1e72953_app1.docx]

Thank you for agreeing to do this interview. It will take about 20–30 minutes, and you will receive $20 for your time. Your feedback is very important to us, as it helps us make the program better. Do you have any questions before we get started?

[**Interviewer obtains verbal consent to begin recording**]

I will ask you a few open-ended questions about your experience. Please share your thoughts in your own words.

Overall Experience

1. What made you decide to join the Empower@Home program?
2. What was your experience like with the program?
3. How did the program meet your needs? Were there any ways it didn’t?
4. What helped you succeed or made it easier for you to participate?
5. Did you face any challenges or obstacles while using the program? What made it harder for you to complete or get the most out of it?

Experience with Coaching

1. How would you describe your relationship and interactions with your coach?
2. How did the coaching sessions fit with the rest of the program? Were there ways they could have been better connected?
3. Did you face any challenges while working with your coach?
4. What made the coaching process easier for you?
5. What about your coach’s style or approach worked really well for you?
6. Was there anything about your coach’s style or approach that didn’t work as well for you?
7. In what ways did your coach support you during the program?
8. How useful was the feedback or guidance your coach gave you on the lessons and exercises?
9. How did the coaching sessions shape your overall experience with the program?
10. If you could change something about the coaching sessions—like the structure, timing, or what they focused on—what would you suggest?
11. Is there anything else you wish your coach had done differently to support you better?

Recommendations

1. What would you tell someone who’s considering participating in this program?
2. If this program were offered as a paid service in the future, how likely would you be to use it or recommend it to others? What factors would influence your decision?
3. What aspects of the program do you think are most valuable and worth paying for? Are there any features or services you think should be added or improved to make it more appealing?
4. Is there anything else you would like to share with me regarding your experience?

Closing

Thank you so much for sharing your thoughts. Your feedback is very valuable to us. I’ll now stop the recording.
