# Supplementary material for: Leveraging Aging Service Providers to Support Internet-Based Cognitive Behavioral Therapy for Depression in Homebound Older Adults: Protocol for a Type 1 Hybrid Effectiveness-Implementation Randomized Controlled Trial
Source: JMIR Res Protoc. 2025 Sep 5;14:e72953. doi: 10.2196/72953 (PMC12449670; doi:10.2196/72953)
Supplement: Multimedia Appendix 3 [file resprot_v14i1e72953_app3.pdf]

SUMMARY STATEMENT
(Privileged Communication)

PROGRAM CONTACT:
Laura Rowland
laura.rowland@nih.gov

Release Date: 04/10/2024
Revised Date:

Application Number: 1R01MH137064-01

Principal Investigator

XIANG, XIAOLING

Applicant Organization: UNIVERSITY OF MICHIGAN AT ANN ARBOR

Review Group: EMHI
Effectiveness of Mental Health Interventions Study Section

Meeting Date: 03/12/2024
Council: MAY 2024
Requested Start: 07/01/2024
Opportunity Number: PAR-21-130
PCC: A4-GPS

Project Title: Community-Embedded, Layperson-Supported Digital Mental Health Intervention for Homebound Older Adults with Depression: A Type 1 Hybrid Effectiveness-Implementation RCT
SRG Action: Impact Score:26
Next Steps: Visit https://grants.nih.gov/grants/next_steps.htm
Human Subjects: 30-Human subjects involved - Certified, no SRG concerns
Animal Subjects: 10-No live vertebrate animals involved for competing appl.
Gender: 1A-Both genders, scientifically acceptable
Minority: 1A-Minorities and non-minorities, scientifically acceptable
Age: 3A-No children included, scientifically acceptable

Project Year	Direct Costs Requested	Estimated Total Cost
1	468,723	734,113
2	439,688	688,639
3	449,257	703,626
4	473,544	741,664
5	349,045	546,674
TOTAL	2,180,257	3,414,715

EARLY STAGE INVESTIGATOR
NEW INVESTIGATOR

1R01MH137064-01 XIANG, XIAOLING

EARLY STAGE INVESTIGATOR
NEW INVESTIGATOR
PROTECTION OF HUMAN SUBJECTS UNACCEPTABLE
COMMITTEE BUDGET RECOMMENDATIONS

RESUME AND SUMMARY OF DISCUSSION: This is a new application in response to Clinical Trials to Test the Effectiveness of Treatment, Preventive, and Services Interventions (R01 Clinical Trial Required) PAR-21-130. The application proposes a randomized Type I hybrid effectiveness-implementation intervention trial to determine the clinical effects of a novel digital mental health intervention, Empower@Home, on depression symptoms measured using the Patient Health Questionnaire-9 (PHQ-9). The study will recruit 256 low-income homebound older adults to Empower@Home or an enhanced usual care. Further, the study will use a mixed-methods approach to examine intervention change mechanisms and identify barriers to implementation. This application provides a clear presentation that possesses several notable strengths including good preliminary data, and a great team and environment. The conceptual model and research methods are good and well supported by the inclusion of a mixed-methods approach. The intervention is cost effective and likely scalable. Despite these strengths, several weaknesses exist. The proposed study was perceived to be ambitious, and not highly innovative. Given what appears to be a small sample for the scope of the project, questions were raised about the generalizability of the intervention and of the findings beyond the product evaluated in the current study. The plan to collect implementation and treatment fidelity data are inadequate. Details related to the primary outcomes, the role of coaches, and rationale for investigator's level of effort are insufficient. Despite these minor weaknesses, this is an excellent application of high significance about which reviewers were very enthusiastic.

DESCRIPTION (provided by applicant): Integrating digital mental health interventions (DMHIs) into aging services agencies offers a scalable and potentially cost-effective approach to mitigating the mental health disparities experienced by homebound older adults. This process involves using aging service providers to deploy DMHIs and assist older adults in initiating and consistently using these digital tools. The long-term goal of the proposed study is to identify the features that define effective DMHIs for homebound older adults and to cultivate sustainable strategies for implementing DMHIs within community-based aging services. The specific aims are to (a) determine the clinical effects of Empower@Home—a novel DMHI explicitly designed for homebound older adults and supported by lay providers within aging service agencies—against enhanced usual care; (b) examine the intervention change mechanisms; and (c) evaluate implementation process by identifying barriers, and facilitators from multi- stakeholder perspectives. Empower@Home applies the established principles of cognitive-behavioral therapy (CBT) to address depressive symptoms prevalent in homebound older adults, enriched with engaging, character-driven storytelling conveyed through short videos. Preliminary research indicates that Empower@Home outperforms similar programs in usability and shows remarkable adherence (90% completion rate) and clinical efficacy when supported by trained research staff. The current project proposes a randomized Type I hybrid effectiveness-implementation intervention trial with 256 low-income homebound older adults served by three aging service agencies in Michigan. Participants will be allocated to receive either Empower@Home supported by agency staff or enhanced usual care. This intervention comprises nine self- help online sessions presented via short videos and text narrations on a dedicated web platform, accompanied by a physical workbook summarizing sessions and featuring worksheets for home exercises, plus weekly coaching calls conducted by trained agency staff. Enhanced usual care encompasses aging services such as case

management and home support, augmented with a handout that includes psychoeducation, local resource guides, and bi-weekly depression assessments. Depression symptoms will be primarily measured using the Patient Health Questionnaire-9 (PHQ-9) at 12-, 24-, and 36-weeks post-baseline, complemented by five in-app assessments during sessions 1, 3, 5, 7, and 9. A combination of methods will examine and explore CBT-associated, engagement-related, and supporter-related change mechanisms. Furthermore, a qualitative assessment of the implementation process will be undertaken, with data being analyzed through traditional qualitative data analysis and natural language processing techniques. It is hypothesized that the treatment will prove superior to the enhanced usual care.

PUBLIC HEALTH RELEVANCE: This study aims to identify a cost-effective and scalable strategy for reducing disparities in access to mental health services among the rapidly growing population of homebound older adults. It plans to evaluate a novel digital mental health intervention for depression, supported by lay providers within aging service agencies, and systematically examine the implementation process, pinpointing barriers, facilitators, and conditions for sustainment from multi-stakeholder perspectives. The expected findings aspire to guide the implementation of accessible, scalable, and cost-effective depression care within aging services, a non-specialty sector with unparalleled reach to the target demographic.

CRITIQUE 1
Significance: 3
Investigator(s): 2
Innovation: 5
Approach: 4
Environment: 1

Overall Impact:
This R01 proposal aims to test the effectiveness of a novel digital mental health intervention, Empower@Home, to provide treatment for depression for homebound older adults. Empower@Home has been previously investigated demonstrating promising impacts of depressive symptoms and feasibility of coach supported model. The current proposal will extend past work by conducting a large effectiveness study (comparing Empower@Home to enhanced usual care) and collect information about implementation potential following the logic of a Type 1 Effectiveness-Implementation Hybrid Trial. Implementation will be supported with coaches who are trained aging service providers. The project comes from a well-experienced investigative team who has collected pilot data to demonstrate the feasibility of Empower@Home. The PI is an early staged investigator who has strong experience in this area piloting work and is supported by a co-I with strong experience running clinical trials in DMHI and an investigative team with the necessary expertise to succeed at this project. The project addresses an important need for scalable and palatable interventions for older adults. The implementation focus including leveraging aging service providers as coaches is a considerable strength, however the implementation considerations in this project are limited to explorations of barriers and facilitators. The Empower@Home program is specifically designed and tailored to address needs and interests of the older adult populations. The application has considerable strengths including a clear conceptual model underlying an experimental therapeutics approach, multi-observer reports of the primary measures, and implementation focus combining qualitative and quantitative evaluation components. The relative contribution following previous work is somewhat incremental and questions whether more considerable focus can be put on implementation aspects, some components of the proposal are underspecified such as the cost-effectiveness analysis, and assessment of the coaching. These elements would be especially useful for informing generalizable knowledge from this project,

rather than the Empower@Home product itself, and the implementation focus of the work. Overall, this is a strong proposal that has some minor weaknesses.

1. Significance:
Strengths
- Depression is a common but treatable concern among homebound older adults.
- Digital mental health interventions represent a scalable and feasible treatment for providing evidence-based interventions for older adults if logistical barriers can be addressed for deployment.
- Leveraging service providers are coaches provides an opportunity to integrate into existing service structures.

Weaknesses
- Rationale for a Type 1 hybrid design versus a design that more formerly tests an implementation strategy (i.e., Type 2 or Type 3) is lacking, this is particularly important given the considerable pilot work already conducted. It raises concerns whether the contribution of the current project is incremental over other knowledge.
- Useful to differentiate what is the generalizable knowledge that will be gained here to inform subsequent interventions that could address depression among homebound adults and implementations of digital mental health interventions more broadly.

2. Investigator(s):
Strengths
- This is a strong early-stage investigator PI who has conducted pilot studies leading to this study, demonstrating feasibility of Empower@Home and experience working with older adult and aging services.
- Strong investigative team includes co-I support in clinical trials for digital mental health and depression, implementation science, data science, and quantitative data analysis.

Weaknesses
- More information could be provided about co-I's Piatt's experience in qualitative data analysis to support that portion of the evaluation.

3. Innovation:
Strengths
- Character-driven storyline brings engagement elements and is evaluated in the quant + qual mechanism evaluation.

Weaknesses
- User-centered design and community participatory design is not innovative.
- Limited innovation in study design or analytic strategy.
- The current project might be an incremental contribution above and beyond further work.

4. Approach:
Strengths
- Coaching follows structured activities based on conceptual model of the efficiency model of support.
- Assessment of depression will include both self-report and clinician-rated measures.
- Assessment measures guided by NIMH common data elements.
- Specified mixed methods QUAL+QUANT approach is a strength for understanding intervention change mechanisms and follows conceptually grounded model of hypothesized change mechanisms.

Weaknesses

- Justification for eligibility criteria of PHQ-9 greater of equal to 8 vs the more traditional PHQ-9 of greater or equal to 10 should be provided.
- Providing users devices that are locked down introduces a potential confound between those users who already have devices and can use them for more purposes. The digital literacy of these non-device owners also likely needs to be considered.
- Unclear if the primary outcome is depression as PHQ-9, depression by HAM-D or composite, combination of the two.
- The sample size justification is based off an effect size d = .69, but better clarification of where that effect size estimate comes from would be useful, it seems to be consistent with past work, but also effect sizes in smaller studies are often not robust to larger investigations. The study might be underpowered on the primary outcome which would also make evaluations of mechanisms and moderators more challenging.
- More information could be provided about how coaching fidelity will be determined (i.e., what factors will be coded, whether this codebook exists, who will be doing the fidelity assessment).
- No consideration of connection or relation to the coach who is supporting the intervention, given this is a coached intervention better understanding the activities and impact of the coaching is an important component.
- Digital literacy is proposed to be measured with the Attitudes Towards Computers scale which assesses perceptions but not technical skills. These skills are especially critical to evaluate in older adults who might have lower levels of digital literacy.
- Cost-effectiveness analysis is under specified, more details can be provided about what costs will be included in the analyses and how.
- Use of NLP for qualitative analysis could require more information supporting the feasibility of this approach vs. human coding or how NLP and human coding will be supplementary.

5. Environment:
Strengths
- Strong environment from the University of Michigan.
- Collaborative partners provide the necessary recruitment and opportunities for scaling and addressing implementation perspective.

Weaknesses
- [None noted]

Study Timeline:
Strengths
- [None noted]

Weaknesses
- More detailed study milestones could be provided including expected rates of enrollment.

Collaboration (Only R01 Collaborative applications in response to PAR-21-129):
- [None noted]

Protections for Human Subjects:
Unacceptable Risks and/or Inadequate Protections.
- It is proposed that participants may be removed for worsening symptoms but the algorithm for determining worsening symptoms is not well-specified.

Data and Safety Monitoring Plan (Applicable for Clinical Trials Only):
Acceptable

Inclusion Plans:

- Sex/Gender: Distribution justified scientifically.
- Race/Ethnicity: Distribution justified scientifically.
- For NIH-Defined Phase III trials, Plans for valid design and analysis: Scientifically acceptable.
- Inclusion/Exclusion Based on Age: Distribution justified scientifically.

Vertebrate Animals:
Not Applicable (No Vertebrate Animals).

Biohazards:
Not Applicable (No Biohazards).

Applications from Foreign Organizations:
Not Applicable (No Foreign Organizations).

Select Agents:
Not Applicable (No Select Agents).

Resource Sharing Plans:
Acceptable

Authentication of Key Biological and/or Chemical Resources:
Not Applicable (No Relevant Resources).

Budget and Period of Support:
Recommend as Requested.

CRITIQUE 2
Significance: 1
Investigator(s): 3
Innovation: 1
Approach: 3
Environment: 1

Overall Impact:
This study tests a novel digital intervention for homebound older adults' mental health. Good preliminary evidence suggests that this intervention may be feasible and effective. The sample size for this project is small, which limits the potential impact of the study. The investigative team has good experience with this intervention but lacks experience in projects of this size.

1. Significance:
Strengths
- Older adults face significant risks for mental health programs. This intervention has the potential to improve their lives and mental health outcomes in a cost-effective manner.
- The intervention is practical and scalable.
- Data will be collected that will inform scalability and replicability in other locations/sites.

Weaknesses
- None noted.

2. Investigator(s):

Strengths
- The team has a strong history of collaboration.
- The PI has strong experience with this intervention, population, and community partners.

Weaknesses
- It is not clear that the team has enough experience with projects of this size and scope.

3. Innovation:
Strengths
- This digital health approach is an innovative solution for older adults who are homebound with the potential to positively impact many people.

Weaknesses
- None noted.

4. Approach:
Strengths
- The RCT design seems appropriate for this stage of this intervention's development.
- The outcome measures are appropriate for this study.
- Community partners have provided input into the intervention and it's feasibility.
- Information will be collected that will inform scalability and implementation elsewhere.

Weaknesses
- Although power analyses support the sample size for the main analyses, the sample seems small for this study's scope.
- The small sample size precludes analyses by subgroups.

5. Environment:
Strengths
- The environment seems appropriate for this project.

Weaknesses
- None noted.

Study Timeline:
Strengths
- The timeline seems appropriate.

Weaknesses
- None noted.

Collaboration (Only R01 Collaborative applications in response to PAR-21-129):
- [None noted]

Protections for Human Subjects:
Acceptable Risks and/or Adequate Protections.
- Acceptable

Data and Safety Monitoring Plan (Applicable for Clinical Trials Only):
Acceptable
- o Acceptable

Inclusion Plans:
- Sex/Gender: Distribution justified scientifically.
- Race/Ethnicity: Distribution justified scientifically.
- For NIH-Defined Phase III trials, Plans for valid design and analysis:

- Inclusion/Exclusion Based on Age: Distribution justified scientifically.
- Acceptable

Vertebrate Animals:
Not Applicable (No Vertebrate Animals).

Biohazards:
Not Applicable (No Biohazards).

Applications from Foreign Organizations:
Not Applicable (No Foreign Organizations).

Select Agents:
Not Applicable (No Select Agents).

Resource Sharing Plans:
Not Applicable (No Relevant Resources).

Authentication of Key Biological and/or Chemical Resources:
Not Applicable (No Relevant Resources).

Budget and Period of Support:
Recommend as Requested.

CRITIQUE 3
Significance: 1
Investigator(s): 1
Innovation: 2
Approach: 3
Environment: 1

Overall Impact:
The long-term goal of the proposed R01 study is to identify the features that define effective digital mental health interventions (DMHIs) for homebound older adults and to cultivate sustainable strategies for implementing DMHIs within community-based aging services. The team proposes testing Empower@Home, a DMHI that applies principles of cognitive-behavioral therapy (CBT) to address depressive symptoms prevalent in homebound older adults, enriched with engaging, character-driven storytelling conveyed through short videos. Preliminary research is highly promising and indicates that Empower@Home outperforms similar programs in usability and shows excellent adherence and clinical efficacy to reduce depression symptoms when supported by trained research staff. The current project proposes a randomized Type I hybrid effectiveness-implementation intervention trial. I am enthusiastic about this application. Significance and innovation are strong. The investigators and environment are outstanding. Scientific rigor of the research approach is demonstrated and supported by strong preliminary research. Weaknesses in the application are minor. Overall, the potential impact is high.

1. Significance:
Strengths

- Depression among the target population of elders who are homebound is a prevalent and serious health problem with high co-morbidity with other disorders and negative effects on quality of life.
- The scientific premise for the intervention is well supported from the literature and the team's preliminary data.
- Scientific rigor of previous work is strong.
- A strong case is made for producing an evidenced-based, accessible DMHI to mitigate depression for elders who are homebound.
- Efficacy data from preliminary work make a compelling case that Empower@Home has strong potential to demonstrate effectiveness and scalability.

Weaknesses

- Efficacy evidence is based on comparison to a control condition rather than another active treatment.

2. Investigator(s):

Strengths

- Dr. Xiang is an outstanding applicant as an early investigator for this R01 application.
- The team has demonstrated strong track records in their research careers and have a. history of successful collaboration.
- Complementary areas of expertise make this an excellent team.

Weaknesses

- Effort for investigators is very limited during the academic year. This effort allocation could limit time for needed study oversight by investigators. This weakness is minor and should be managed by the investigators to balance time as a team devoted to the research and other work demands.

3. Innovation:

Strengths

- Using storyline "entertainment' via a character story to engage participants is innovative. This approach was informed by prior work with stakeholder input.
- Using existing elder services staff and infrastructure for delivery is rather novel and illustrates the team's community engaged approach and project scalability potential.
- Real world intervention delivery and testing via existing elder services boosts evidence for real world effectiveness.

Weaknesses

- Other DMHIs to mitigate depression exist, so use of a digital approach is not innovative in itself. However, the investigators compared their application (app) to existing apps and found it superior. They also adapted the digital approach to match needs of their target population.
- Specifically, description of the treatment strategies to reduce cognitive arousal, the key mechanism identified in sleep disturbance, are not adequately explicated.

4. Approach:

Strengths

- Preliminary studies provide robust support for the intervention and mesarch approach.
- Usability ratings, acceptability, and efficacy results from prior intervention testing are strong in comparison to other apps. These results are impressive.
- The PI has experience with many components of the design from prior research. Notably, experience with recruitment, data collection, and intervention delivery inform this application and provide strong support for likelihood of success.
- Staff and agency training approaches are well described and tested.

- Measures are reliable and valid.

Weaknesses

- Multiple measures are proposed but not all are administered multiple times; most are done at T1 and T2, and burden is addressed; this is a minor concern.
- Use of NLP is mentioned toward the end of the application but seems like a “nice to have” vs. a “need to have” approach to enhance the qualitative analysis.
- The multiple components of the approach are ambitious.
- Availability of technical support is not addressed.

5. Environment:

Strengths

- The universities and clinical partners are outstanding.
- Excellent resources exist to support the research and to support recruitment.
- Letters of support confirm institutional/clinical agency commitment.

Weaknesses

- None noted.

Study Timeline:

Strengths

- Investigators describe potential challenges and pose solutions.
- The timeline outlines major activities clearly with a good plan.

Weaknesses

- The plan is ambitious but not unrealistic given the preliminary data, experience, and resources.

Collaboration (Only R01 Collaborative applications in response to PAR-21-129):

Adequate

- Not applicable.

Protections for Human Subjects:

Acceptable Risks and/or Adequate Protections.

- Careful consideration of risk assessment and follow-up as needed.

Data and Safety Monitoring Plan (Applicable for Clinical Trials Only):

Acceptable

- Detailed, appropriate DSMP plan is presented.

Inclusion Plans:

- Sex/Gender: Distribution justified scientifically.
- Race/Ethnicity: Distribution justified scientifically.
- For NIH-Defined Phase III trials, Plans for valid design and analysis: Not applicable.
- Inclusion/Exclusion Based on Age: Distribution justified scientifically.
  - Are there HIPAA consideration about inclusion of persons with very high age re: possible ability to identify? This earlier concern may have been dropped as many more people are living to 90+ and even 100+.

Vertebrate Animals:

Not Applicable (No Vertebrate Animals).

Biohazards:

Not Applicable (No Biohazards).

Applications from Foreign Organizations:
Not Applicable (No Foreign Organizations).

Select Agents:
Not Applicable (No Select Agents).

Resource Sharing Plans:
Not Applicable (No Relevant Resources).

Authentication of Key Biological and/or Chemical Resources:
Not Applicable (No Relevant Resources).

Budget and Period of Support:
Recommend as Requested.
Recommended budget modifications or possible overlap identified:

- Consider spreading more effort over the academic year.

THE FOLLOWING SECTIONS WERE PREPARED BY THE SCIENTIFIC REVIEW OFFICER TO SUMMARIZE THE OUTCOME OF DISCUSSIONS OF THE REVIEW COMMITTEE, OR REVIEWERS' WRITTEN CRITIQUES, ON THE FOLLOWING ISSUES:

PROTECTION OF HUMAN SUBJECTS: UNACCEPTABLE.
The algorithm for determining participants who will be removed due to worsening symptoms is not well-specified.

INCLUSION OF WOMEN PLAN: ACCEPTABLE

INCLUSION OF MINORITIES PLAN: ACCEPTABLE

INCLUSION ACROSS THE LIFESPAN: ACCEPTABLE

COMMITTEE BUDGET RECOMMENDATIONS: Effort allocation during the academic year seems low. Investigators might consider spreading more effort over the academic year.

Footnotes for 1R01MH137064-01; PI Name: Xiang, Xiaoling

NIH has modified its policy regarding the receipt of resubmissions (amended applications).See Guide Notice NOT-OD-18-197 at https://grants.nih.gov/grants/guide/notice-files/NOT-OD-18-197.html. The impact/priority score is calculated after discussion of an application by averaging the overall scores (1-9) given by all voting reviewers on the committee and multiplying by 10. The criterion scores are submitted prior to the meeting by the individual reviewers assigned to an application, and are not discussed specifically at the review meeting or calculated into the overall impact score. Some applications also receive a percentile ranking. For details on the review process, see
http://grants.nih.gov/grants/peer_review_process.htm#scoring.

MEETING ROSTER

Effectiveness of Mental Health Interventions Study Section
National Institute of Mental Health Initial Review Group
NATIONAL INSTITUTE OF MENTAL HEALTH
EMHI
03/12/2024 - 03/13/2024

Notice of NIH Policy to All Applicants: Meeting rosters are provided for information purposes only. Applicant investigators and institutional officials must not communicate directly with study section members about an application before or after the review. Failure to observe this policy will create a serious breach of integrity in the peer review process, and may lead to actions outlined in NOT-OD-22-044 at https://grants.nih.gov/grants/guide/notice-files/NOT-OD-22-044.html, including removal of the application from immediate review.

CHAIRPERSON(S)

SALZER, MARK S, PHD
PROFESSOR
DEPARTMENT OF SOCIAL AND BEHAVIORAL SCIENCES
COLLEGE OF PUBLIC HEALTH
TEMPLE UNIVERSITY
PHILADELPHIA, PA 19122

MEMBERS

ANDOVER, MARGARET S, PHD
PROFESSOR
DEPARTMENT OF PSYCHOLOGY
FORDHAM UNIVERSITY
BRONX, NY 10458

BAUMGARTNER, JOY NOEL, PHD *
ASSOCIATE PROFESSOR
SCHOOL OF SOCIAL WORK
UNIVERSITY OF NORTH CAROLINA
CHAPEL HILL, NC 27599

BEARMAN, SARAH KATE, PHD
ASSOCIATE PROFESSOR
DEPARTMENT OF EDUCATIONAL PSYCHOLOGY
COLLEGE OF EDUCATION
THE UNIVERSITY OF TEXAS AT AUSTIN
AUSTIN, TX 78712

BENITO, KRISTEN G, PHD *
ASSOCIATE PROFESSOR
DEPARTMENT OF PSYCHIATRY AND HUMAN BEHAVIOR
ALPERT MEDICAL SCHOOL
BROWN UNIVERSITY
PROVIDENCE, RI 02912

BOYD, RHONDA C, PHD
ASSOCIATE PROFESSOR
DEPARTMENT OF CHILD AND ADOLESCENT PSYCHIATRY AND BEHAVIORAL SCIENCES
CHILDREN'S HOSPITAL OF PHILADELPHIA
UNIVERSITY OF PENNSYLVANIA SCHOOL OF MEDICINE
PHILADELPHIA, PA 19146

BREITENSTEIN, SUSAN M, PHD *
ASSOCIATE PROFESSOR
COLLEGE OF NURSING
THE OHIO STATE UNIVERSITY
COLUMBUS, OH 43210

BUSTAMANTE, EDUARDO ESTEBAN, PHD *
ASSISTANT PROFESSOR
DEPARTMENT OF KINESIOLOGY AND NUTRITION
UNIVERSITY OF ILLINOIS AT CHICAGO
CHICAGO, IL 60607

CHACKO, ANIL, PHD
ASSOCIATE PROFESSOR
DEPARTMENT OF APPLIED PSYCHOLOGY
STEINHARDT SCHOOL OF CULTURE, EDUCATION AND HUMAN DEVELOPMENT
NEW YORK UNIVERSITY
NEW YORK, NY 10003

CZYZ, EWA KARINA, PHD *
ASSISTANT PROFESSOR
DEPARTMENT OF PSYCHIATRY
UNIVERSITY OF MICHIGAN
ANN ARBOR, MI 48109

DEPP, COLIN A, PHD
PROFESSOR IN RESIDENCE
DEPARTMENT OF PSYCHIATRY
SCHOOL OF MEDICINE
UNIVERSITY OF CALIFORNIA, SAN DIEGO
LA JOLLA, CA 92093

DOTSON, VONETTA M, PHD *
ASSOCIATE PROFESSOR
DEPARTMENT OF PSYCHOLOGY
GEORGIA STATE UNIVERSITY
ATLANTA, GA 30302

FORD, JULIAN D, PHD *
PROFESSOR
DEPARTMENT OF PSYCHIATRY
SCHOOL OF MEDICINE
UNIVERSITY OF CONNECTICUT
FARMINGTON, CT 06030

GOLTZ, HEATHER HONORE, PHD *
ASSISTANT PROFESSOR
SOCIAL WORK PROGRAM
COLLEGE OF PUBLIC SERVICE
UNIVERSITY OF HOUSTON-DOWNTOWN
HOUSTON, TX 77002

GRAY, CHRISTINE L, MPH, PHD *
ASSISTANT RESEARCH PROFESSOR
CENTER FOR HEALTH POLICY AND INEQUALITIES RESEARCH
DUKE UNIVERSITY
DURHAM, NC 27710

HILL, RYAN, PHD *
ASSISTANT PROFESSOR
DEPARTMENT OF PSYCHOLOGY
LOUISIANA STATE UNIVERSITY
BATON ROUGE, LA 70802

HOROWITZ, JUNE ANDREWS, PHD *
PROFESSOR AND ASSOCIATE DEAN GRADUATE PROGRAMS AND RESEARCH
COLLEGE OF NURSING
UNIVERSITY OF MASSACHUSETTS DARTMOUTH
DARTMOUTH, MA 02747

LEAVER, AMBER MICHELLE, PHD *
RESEARCH ASSOCIATE PROFESSOR
DEPARTMENT OF RADIOLOGY
BASIC AND TRANSLATIONAL RADIOLOGY RESEARCH
FEINBERG SCHOOL OF MEDICINE
NORTHWESTERN UNIVERSITY
CHICAGO, IL 60611

LEVINSON, CHERI ALICIA, PHD *
ASSOCIATE PROFESSOR
DEPARTMENT OF PSYCHOLOGY
UNIVERSITY OF LOUISVILLE
LOUISVILLE, KY 40292

MASIA, CARRIE L, PHD *
PROFESSOR
DEPARTMENT OF PSYCHOLOGY
COLLEGE OF HUMANITIES AND SOCIAL SCIENCES
MONTCLAIR STATE UNIVERSITY
MONTCLAIR, NJ 07043

MORGAN-LOPEZ, ANTONIO A, PHD
FELLOW IN QUANTITATIVE PSYCHOLOGY
RISK BEHAVIOR AND FAMILY RESEARCH PROGRAM
RTI INTERNATIONAL
RESEARCH TRIANGLE PARK, NC 27709

MUSSER, ERICA D, PHD *
ASSOCIATE PROFESSOR
DEPARTMENT OF PSYCHOLOGY
FLORIDA INTERNATIONAL UNIVERSITY
MIAMI, FL 33199

NADEEM, ERUM, PHD *
ASSOCIATE PROFESSOR
DEPARTMENT OF SCHOOL PSYCHOLOGY
RUTGERS UNIVERSITY
PISCATAWAY, NJ 08854

NARR, KATHERINE L, PHD *
PROFESSOR
DEPARTMENT OF NEUROLOGY, PSYCHIATRY AND BIOBEHAVIORAL SCIENCE
DAVID GEFFEN SCHOOL OF MEDICINE
UNIVERSITY OF CALIFORNIA, LOS ANGELES
LOS ANGELES, CA 90095

NGUYEN, AMANDA J, PHD *
ASSOCIATE PROFESSOR
DEPARTMENT OF HUMAN SERVICES
UNIVERSITY OF VIRGINIA
EARLYSVILLE, VA 22936

PAVAO, CARLOS A. O., DRPH
CLINICAL ASSOCIATE PROFESSOR
HEALTH POLICY AND BEHAVIORAL SCIENCES
SCHOOL OF PUBLIC HEALTH
GEORGIA STATE UNIVERSITY
ATLANTA, GA 30303

PAVLICOVA, MARTINA, PHD *
ASSOCIATE PROFESSOR
DEPARTMENT OF BIOSTATISTICS
MAILMAN SCHOOL OF PUBLIC HEALTH
COLUMBIA UNIVERSITY
NEW YORK, NY 10032

RHOADES, GALENA, PHD *
RESEARCH PROFESSOR
DEPARTMENT OF PSYCHOLOGY
UNIVERSITY OF DENVER
DENVER, CO 80208

RUBLE, LISA A, PHD
EARL F. SMITH DISTINGUISHED PROFESSOR IN SPECIAL EDUCATION AND AUTISM
DEPARTMENT OF SPECIAL EDUCATION
BALL STATE UNIVERSITY
MUNCIE, IN 47306

RUGGIERO, KENNETH J, PHD *
PROFESSOR
TECHNOLOGY APPLICATION CENTER FOR HEALTHFUL LIFESTYLES
COLLEGE OF NURSING
MEDICAL UNIVERSITY OF SOUTH CAROLINA
CHARLESTON, SC 29425

SCHUELLER, STEPHEN, PHD
ASSOCIATE PROFESSOR
DEPARTMENT OF PSYCHOLOGICAL SCIENCE
UNIVERSITY OF CALIFORNIA, IRVINE
IRVINE, CA 92697

SHAFFER, ANNE ELIZABETH, PHD *
ASSOCIATE PROFESSOR
DEPARTMENT OF PSYCHOLOGY
UNIVERSITY OF GEORGIA
ATHENS, GA 30602

SILLER, MICHAEL, PHD *
ASSOCIATE PROFESSOR
DEPARTMENT OF EDUCATIONAL PSYCHOLOGY
UNIVERSITY OF NORTH TEXAS
DENTON, TX 76203

STOVER, CARLA S, PHD *
ASSOCIATE PROFESSOR
CHILD STUDY CENTER
SCHOOL OF MEDICINE
YALE UNIVERSITY
NEW HAVEN, CT 06520

STUDTS, CHRISTINA RUTH, PHD *
ASSOCIATE PROFESSOR
DEPARTMENT OF PEDIATRICS
UNIVERSITY OF COLORADO ANSCHUTZ MEDICAL CAMPUS
AURORA, CO 80045

SYLVIA, LOUISA, PHD
ASSOCIATE PROFESSOR/PSYCHOLOGIST
DAUTEN FAMILY CENTER
MASSACHUSETTS GENERAL HOSPITAL
DEPARTMENT OF PSYCHIATRY
HARVARD MEDICAL SCHOOL
BOSTON, MA 02114

WAINER, ALLISON LEIGH, PHD *
ASSOCIATE PROFESSOR
DEPARTMENT OF PSYCHIATRY AND BEHAVIORAL SCIENCES
RUSH UNIVERSITY MEDICAL CENTER
CHICAGO, IL 60612

WEERSING, V ROBIN, PHD
PROFESSOR
DEPARTMENT OF PSYCHOLOGY
SAN DIEGO STATE UNIVERSITY
SAN DIEGO, CA 92120

WHITTON, SARAH W, PHD *
PROFESSOR
DEPARTMENT OF PSYCHOLOGY
UNIVERSITY OF CINCINNATI
CINCINNATI, OH 45227

WILCOX, HOLLY C, PHD
PROFESSOR
DEPARTMENT OF MENTAL HEALTH
JOHNS HOPKINS BLOOMBERG SCHOOL OF PUBLIC HEALTH
BALTIMORE, MD 21205

WOLITZKY-TAYLOR, KATE BASIA, PHD *
ASSOCIATE PROFESSOR
DEPARTMENT OF PSYCHIATRY AND BIOBEHAVIORAL SCIENCE
DAVID GEFFEN SCHOOL OF MEDICINE
UNIVERSITY OF CALIFORNIA, LOS ANGELES
LOS ANGELES, CA 90025

XIAO, YUNYU, PHD *
ASSISTANT PROFESSOR
DEPARTMENT OF POPULATION HEALTH SCIENCES
WEILL MEDICAL COLLEGE OF CORNELL UNIVERSITY
NEW YORK, NY 10065

SCIENTIFIC REVIEW OFFICER

ORTIZ, CLAUDIO DARIO
SCIENTIFIC REVIEW OFFICER
REVIEW BRANCH
DIVISION OF EXTRAMURAL ACTIVITIES
NATIONAL INSTITUTE OF MENTAL HEALTH
NATIONAL INSTITUTES OF HEALTH
BETHESDA, MD 20892

EXTRAMURAL SUPPORT ASSISTANT

KEES, CHRISTOPHER A
EXTRAMURAL SUPPORT ASSISTANT
DIVISION OF EXTRAMURAL ACTIVITIES
NATIONAL INSTITUTE OF MENTAL HEALTH
NATIONAL INSTITUTES OF HEALTH
BETHESDA, MD 20892

* Temporary Member. For grant applications, temporary members may participate in the entire meeting or may review only selected applications as needed.

Consultants are required to absent themselves from the room during the review of any application if their presence would constitute or appear to constitute a conflict of interest.
